# Iatrogenic Duodenal Perforation Treated with Endoscopic Placement of Metallic Clips: A Case Report

**DOI:** 10.1155/2012/609750

**Published:** 2012-02-12

**Authors:** Missale Solomon, Alexander Schlachterman, Ricardo Morgenstern

**Affiliations:** Department of Gastroenterology, Drexel University College of Medicine, Philadelphia, PA 19107, USA

## Abstract

Perforation is one of the major complications encountered during endoscopic procedures. The standard of care for these complications is either surgical intervention or nonoperative medical approach with antibiotics and bowel rest with or without parenteral alimentation. Metallic clips, initially developed to secure hemostasis in bleeding, have been successfully used to close perforations in the gastrointestinal tract (GI) including the duodenum. This avoids perioperative morbidities associated with surgical intervention while limiting the leakage of intestinal contents and peritoneal contamination that is possible with medical management. We present a case of a patient with a lateral duodenal perforation during an endoscopic retrograde cholangiopancreatography (ERCP) which was successfully treated with immediate placement of metallic endoclips.

## 1. Case Report

**Figure 1:**
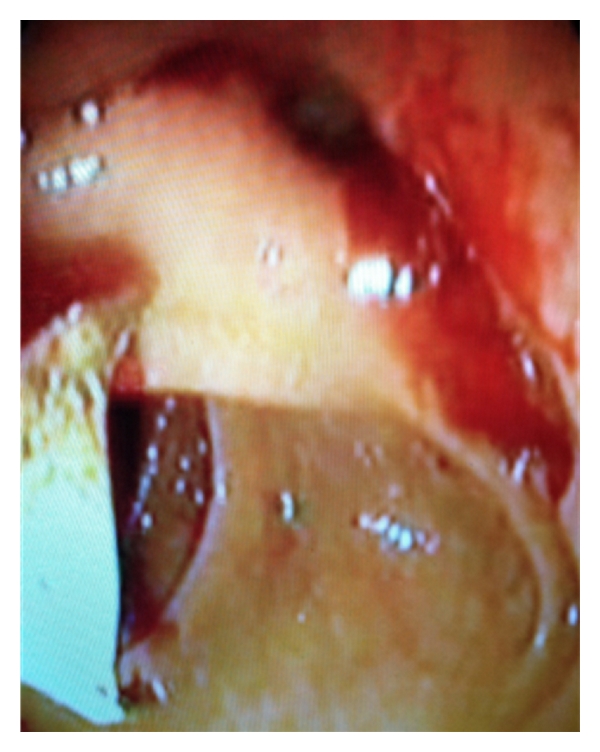
Duodenal perforation visible in upper field prior to first metallic clip placement.

**Figure 2:**
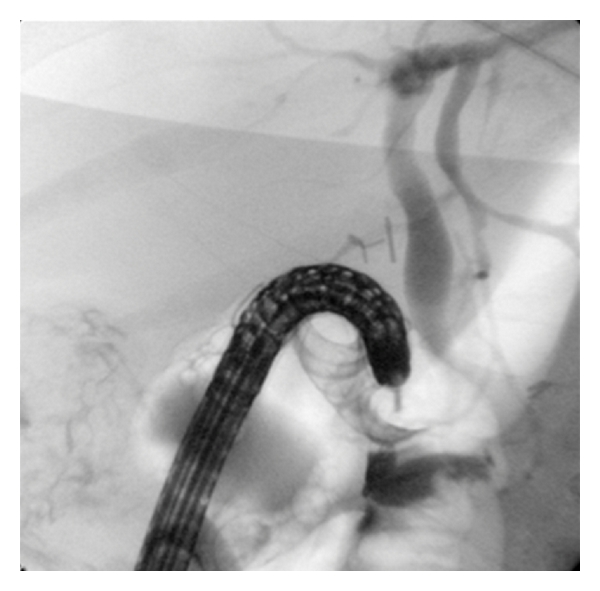
Fluoroscopy showed free air in the peritoneal cavity and peritoneal leakage of contrast injected into the duodenum confirmed a leak.

**Figure 3:**
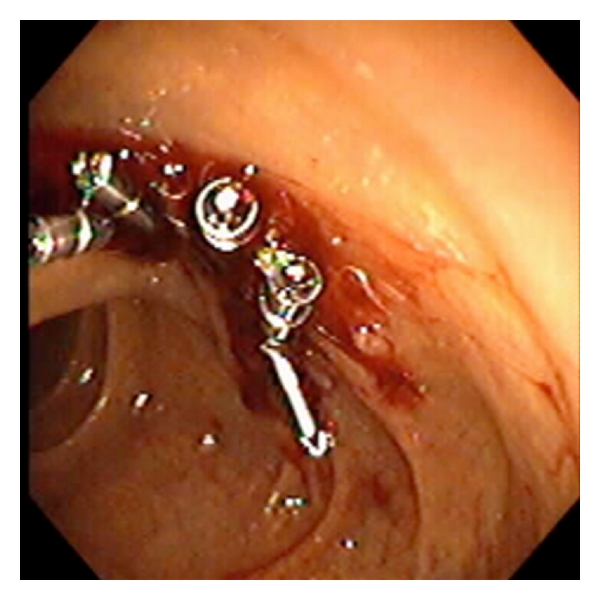
Multiple endoclips have been successfully utilized to close the perforation.

 A 50-year-old woman who underwent pancreatico-jejunostomy nine years prior for chronic pancreatitis was hospitalized with acute relapsing pancreatitis (ARP). Magnetic resonance cholangiopancreatography (MRCP) revealed a dilated pancreatic duct (PD), and subsequently an ERCP was performed. After passage of the side-viewing endoscope to the ampulla, the papilla was examined at which time bleeding was seen coming from the duodenal wall opposite the papilla, (Figure 1). Examination of this area revealed a 1 cm defect compatible with perforation likely caused by the shaft of the endoscope in the presence of a fixed duodenum from her previous surgery. Fluoroscopy showed free air in the peritoneal cavity, and peritoneal leakage of contrast injected into the duodenum confirmed a leak, (Figure 2). The side-viewing endoscope was then exchanged for a gastroscope, and the perforation was closed with 8 hemostatic endoclips (Boston Scientific, Resolution), (Figure 3). Subsequent contrast injection into duodenum showed no leak. The patient consented to conservative treatment of the perforation and was started on broad-spectrum antibiotics, and nasogastric tube was placed. A follow-up upper GI series was normal and CT of the abdomen 2 weeks later showed persistent free air in the peritoneum. Initiation of feeding precipitated an attack of ARP which was managed with total parenteral nutrition (TPN) and later placement of jejunal feeding tube. She was discharged pain-free and stabled on tube feeding after 4 weeks. The patient was subsequently seen in the outpatient clinic without noted complications related to the perforation.

## 2. Discussion

 Iatrogenic duodenal perforations depending on the size and location of the defect are generally managed either with immediate laparatomy and closure or medically with observation and administration of antibiotics [1–3]. In the past two decades, an innovative approach that employs endoscopy in conjunction with IV antibiotics and bowel rest has emerged. To date, several cases have been reported where endoclips have been successfully utilized to close perforations caused by endoscopy [3–9] and ERCP [1, 9].

 The first two cases of metallic clips were reported to close duodenal perforations that occurred during an endoscopic mucosal resection (EMR) of small duodenal tumors. In both cases, the perforations were recognized and treated immediately [10]. Later, closure of postsphincterotomy duodenal perforation and tear during placement of biliary stent was reported, respectively. The inability to use the endoclipping device with a side-viewing endoscope was cited as a drawback in these cases [5, 6]. The repair of a 5 mm duodenal perforation following a snare polypectomy was reported using a total of 5 clips and using of endoclips to close small duodenal perforations less than 10 mm if recognized immediately was recommended [2]. During the same year others gave similar recommendations while reporting on the successful use of a clipping device to treat a duodenal perforation that occurred during an EUS procedure [7, 11]. Unlike the more common spontaneous perforation resulting from peptic ulcer disease, iatrogenic perforations have a relatively lower chance of bacterial contamination due to the fasting patient. This has allowed an opportunity to manage these patients by nonsurgical means.

Successful endoscopic closure of a sphincterotomy-related duodenal perforation was reported in 2005. This study concluded that when endoscopic sphincterotomy causes perforations, endoscopic closure of the defect using metallic clips might be the first choice [4]. Similar findings showed the use of endoclips to close a sphincterotomy-related perforation at the ampulla [8]. A porcine study supports endoscopic closure of duodenal perforations by using the over-the-scope clip (OTSC) system and has identified it to be comparable with surgical closure in a nonsurvival porcine model. At necropsy, all OTSC and surgical closures demonstrated complete sealing of duodenotomy sites [12]. Data from case reports and porcine study seems to suggest that retroperitoneal perforations that are less than 10 mm in a stable patient can be treated with metallic clips employed endoscopically if detected early. However, our patient had perforation of the lateral duodenal wall which has a worse prognosis as compared to sphincterotomy related retroperitoneal perforations. Moreover, her history of prior surgery is an unfavorable prognostic factor as patients with altered foregut anatomy are at increased risk of perforation [3].

The deployment requires significant experience and an endoscopy team familiar to endoclipping device. Moreover, these patients do need to be admitted for observation and intravenous antibiotics with bowel rest and nasogastric drainage when necessary. A surgical consult should be obtained in all these patients as some might possibly go on to develop generalized peritonitis and sepsis.
